# Foodborne Disease Prevention and Broiler Chickens with Reduced *Campylobacter* Infection

**DOI:** 10.3201/eid1903.111593

**Published:** 2013-03

**Authors:** Simon Bahrndorff, Lena Rangstrup-Christensen, Steen Nordentoft, Birthe Hald

**Affiliations:** Author affiliations: Technical University of Denmark, Aarhus, Denmark (S. Bahrndorff, L. Rangstrup-Christensen, S. Nordentoft, B Hald);; Technical University of Denmark, Mørkhøj, Denmark (S. Bahrndorff, S. Nordentoft, B Hald);; National Veterinary Institute, Uppsala, Sweden (L. Rangstrup-Christensen)

**Keywords:** flies, intervention studies, insect vectors, Campylobacter spp., poultry, gastroenteritis, campylobacteriosis, bacteria, fly screens, broiler chickens, Denmark

## Abstract

Studies have suggested that flies play a linking role in the epidemiology of *Campylobacter* spp. in broiler chickens and that fly screens can reduce the prevalence of *Campylobacter* spp. We examined the year-round and long-term effects of fly screens in 10 broiler chicken houses (99 flocks) in Denmark. Prevalence of *Campylobacter* spp.–positive flocks was significantly reduced, from 41.4% during 2003–2005 (before fly screens) to 10.3% in 2006–2009 (with fly screens). In fly screen houses, *Campylobacter* spp. prevalence did not peak during the summer. Nationally, prevalence of *Campylobacter* spp.–positive flocks in Denmark could have been reduced by an estimated 77% during summer had fly screens been part of biosecurity practices. These results imply that fly screens might help reduce prevalence of campylobacteriosis among humans, which is closely linked to *Campylobacter* spp. prevalence among broiler chicken flocks.

*Campylobacter* spp. is the most common cause of enteritis in humans in the European Union; 190,566 cases were reported in 2008 (*1*). However, it has been estimated that only 2.1% of all cases are reported and that in the European Union the true incidence of campylobacteriosis is ≈9 million cases per year (*2*). From 2008 through 2009, the number of human infections in the European Union increased 4%, although there was no statistically significant trend from 2005 through 2009 (*1*). The incidence of campylobacteriosis seems to differ among European countries (*3*). In addition, campylobacteriosis and its sequelae are calculated to cost 0.35 million disability-adjusted life-years per year, totaling €2.4 billion per year (*2*).

Campylobacteriosis is largely perceived to be a foodborne disease. Poultry meat is considered the primary source, causing 20%–30% of all cases; and 50%–80% of all cases might be attributed to the chicken reservoir as a whole (*2*). The incidence of campylobacteriosis cases among humans has been shown to correlate with the prevalence of *Campylobacter* spp. among broiler chickens (*4*).The prevalence of *Campylobacter* spp. in broiler chicken batches varies considerably between EU countries; in 2008, prevalence ranged from 2% to 100% (average 71%) (*5*). Therefore, an international priority for ensuring food safety is the elimination of *Campylobacter* spp. from broiler chicken flocks (*6*,*7*). However, even strict compliance with all biosecurity regulations has failed to control infections in broiler chicken houses during peak months in the summer, indicating that transmission routes, and the blocking of these routes, remain to be fully elucidated and understood.

Studies have repeatedly suggested that flies play a linking role in the epidemiology of *Campylobacter* spp. infections by transmitting *Campylobacter* spp. from fecal sources to poultry (*8*–*10*). Moreover, seasonality of infections in humans (*11*) and broiler chicken flocks (*3*,*4*,*12*,*13*) is similar in northern climates; prevalence peaks during the summer, as does abundance of flies (*11*,*14*). In addition, studies have shown that flies can carry *Campylobacter* spp. under natural conditions (*9*,*15*,*16*) and that hundreds of flies per day pass through ventilation inlets into broiler chicken houses (*15*,*17*). The fly that has been found to most often carry *Campylobacter* spp. is the housefly (*Musca domestica*) (*15*). The retention of *Campylobacter* spp. in this species of fly has been found to be relatively short (*18*). Altogether, these findings suggest that flies could explain some aspects of *Campylobacter* spp. epidemiology.

This association between flies and *Campylobacter* spp. is not surprising because flies are natural carriers of many pathogens, including viruses, fungi, bacteria, and parasites (*9*,*16*,*19*–*21*). Studies have shown that different fly species can harbor up to 100 species of pathogenic microorganisms and that bacteria alone are linked to >65 diseases in humans and animals (*21*–*23*). Houseflies live in close association with humans and breed in animal manure, human excrement, garbage, animal bedding, and decaying organic matter where bacteria are also abundant (*24*). Houseflies have been suggested to be vectors of bacteria, such as *Shigella* spp., *Vibrio cholerae*, *Escherichia coli*, *Aeromonas caviae*, and *Campylobacter* spp. (*15*,*25*–*29*).

To test the hypothesis that the influx of flies increases transmission of *Campylobacter* spp. to broiler chickens during the summer, Hald et al. mounted fly screens on 20 broiler chicken houses in Denmark during the summer (June–October) of 2006 (*30*), when the number of *Campylobacter* spp.–positive flocks in Denmark peaks (*4*). The outcome was a statistically significant decrease, from 51.4% to 15.4%, of *Campylobacter* spp.–positive flocks in the fly-screen houses, whereas prevalence for control houses remained unchanged before and after the intervention (51.7% and 51.4%, respectively). During the summer of 2008, the effect of fly screens was also tested on farms in Iceland where prevalence rates of *Campylobacter* spp. among flocks had been high (*31*). That study found a reduction from 48.3% to 25.6% among flocks in 19 houses from one broiler chicken company and from 31.3% to 17.2% in 16 houses from another company. These published results of the fly screen intervention have covered only the summer and only 1 season.

According to the scientific opinion on *Campylobacter* in broiler chicken meat production, published by the European Food Safety Authority Panel on Biological Hazard (*2*), high priority has been given to generating solid long-term data on biosecurity interventions, including the effect of hygiene barriers and fly screens, as a way to reduce prevalence of *Campylobacter* spp. among flocks of broiler chickens (hereafter referred to as flock prevalence) (*2*). Our aim, therefore, was to generate year-round and long-term data on the effect of fly screen interventions. We present 4 years of data (2006–2009) on the long-term effect of fly screens on *Campylobacter* spp. prevalence among broiler chicken flocks.

## Materials and Methods

### Study Houses

This study was conducted at 10 fly-screened broiler chicken houses situated on 2 one-house farms and 4 two-house farms in Jutland, Denmark. The houses were part of a previous intervention study by Hald et al., conducted in the summer of 2006, in which standard Phiferglass insect screening (Phifer Incorporated, Tuscaloosa, AL, USA) of 18 × 16 mesh/inch^2^ had been installed on 20 broiler chicken houses, thereby excluding 95% of all flies from each house (*30*). The remaining 5% of flies were either so tiny that they were able to penetrate the mesh, or they (and larger flies) could enter the house through open gates or doors during stocking of new chicks (*15*). In addition, 10 control houses that were also part of the study by Hald et al. (*30*) were matched and included for comparison in our study. The criteria used to choose houses are described by Hald et al. (*30*). The houses that were chosen were representative in construction and ventilation type of at least 90% of the broiler chicken houses in Denmark (Technical Appendix). The houses were equipped with fly screens by June 1, 2006, and data were subsequently obtained through 2009.

### *Campylobacter* spp. Flock Prevalence 

Data on *Campylobacter* spp. prevalence among flocks from the 10 houses with fly screens (fly screen houses) during 2006–2009 were compared with data for the same houses during 2003–2005 (before fly screens) and for the 10 control houses (without fly screens) in both periods. In addition, the historical national *Campylobacter* spp. flock prevalence for the 2 periods (2003–2005 and 2006–2009) were included for comparison and are hereafter referred to as national prevalence.

Flock prevalence data were obtained from the national surveillance database (*32*). Since 1998, all broiler chicken flocks in Denmark have been tested for *Campylobacter* spp., and the prevalence of positive flocks has been recorded (*33*). From each flock, 10 pooled cloacal swab samples are obtained at slaughter and analyzed for *Campylobacter* spp. by using a genus-specific PCR (*34*), and results have been collected in the national surveillance database. Data extracted from our study included *Campylobacter* spp. status at slaughter. For flocks that were thinned (part of the flock slaughtered before the end of the rearing period) (*2*), only results from the first slaughter batch were included. 

### Statistical Analyses

Prevalence was calculated as the percentage of flocks positive by 10 pooled cloacal swab samples at slaughter. The Yates χ^2^ test was used to test for differences in *Campylobacter* spp. prevalence, depending on years and treatments. This test was used because of the large sample size of the flocks. Furthermore, odds ratios (ORs) and 95% CIs were calculated. The population attributable fraction (PAF) was calculated according to the method of Webb and Bain (*35*).

## Results

### *Campylobacter* spp. Prevalence during Summer

**Figure 1:**
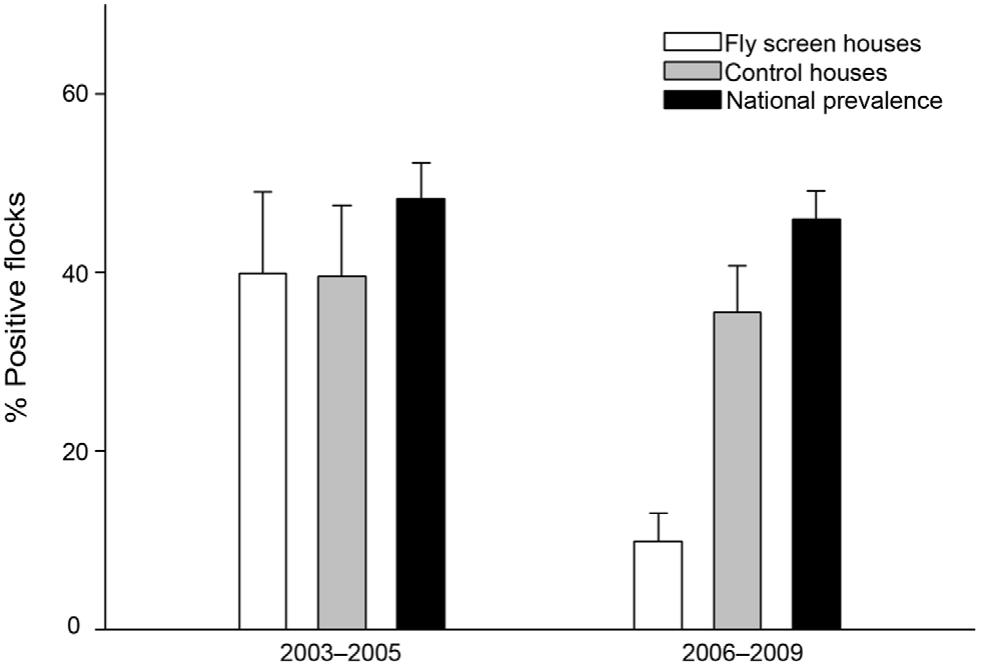
Mean percentage of broiler chicken flocks that were *Campylobacter* spp. positive during summers of 2003–2005 (before fly screens) and 2006–2009 (with fly screens). Prevalence is based on data from June through October each year. Error bars indicate upper limit of SE.

*Campylobacter* spp. prevalence among flocks from fly screen houses decreased significantly from 41.4% in 2003–2005 (before fly screens) to 10.3% in 2006–2009 (with fly screens) (p<0.001; OR 6.1; 95% CI 3.1–12.4), whereas the prevalence reduction in the control houses was minor (not significant), from 41.8% in 2003–2005 to 36.0% in 2006–2009 (p = 0.454; OR 1.3; 95% CI 0.7–2.1) (Figure 1). In comparison, national prevalence, obtained from the surveillance data, decreased significantly from 48.6% to 45.6% during 2003–2005 and 2006–2009 (p<0.001; OR 1.1; 95% CI 1.1–1.2). Prevalence rates of *Campylobacter* spp.–positive flocks for the 3 study groups during the summers of 2003–2005 and 2006–2009 are shown in the Table.

**Table:** *Campylobacter* spp.–positive and –negative broiler chicken flocks in summer (June to October), Denmark

Source	2003–2005				2006–2009			Odds ratio (95% CI)
	No. (%) positive	No. negative	95% CI*		No. (%) positive	No. negative	95% CI	
Fly screen houses	41 (41.4)	58	32.2–51.3		13 (10.3)	113	6.1–16.9	6.1 (3.1–12.4)*
Control houses	41 (41.8)	57	32.6–51.7		48 (36.0)	85	28.4–44.5	1.3 (0.7–2.1)
National prevalence	3,209 (48.6)	3,396	47.4–49.8		3,744 (45.6)	4,471	44.5–46.7	1.1 (1.1–1.2)*

*Significantly different from 1, p<0.001.

Before the fly screen intervention (2003–2005), *Campylobacter* spp. prevalence did not differ between the fly screen houses and the control houses (p = 0.920) or from the national prevalence for the same period (p = 0.188) (Figure 1; Table). During 2003–2005, prevalence for the control houses did not differ from national prevalence (p = 0.221). In contrast, during the period with the intervention (2006–2009), prevalence for fly screen houses was significantly lower than that for the control houses (p<0.001) and lower than national prevalence (p<0.001). During the same period, prevalence was lower for the control houses than nationally (p = 0.036).

### *Campylobacter* spp. Prevalence Seasonal Trends

**Figure 2:**
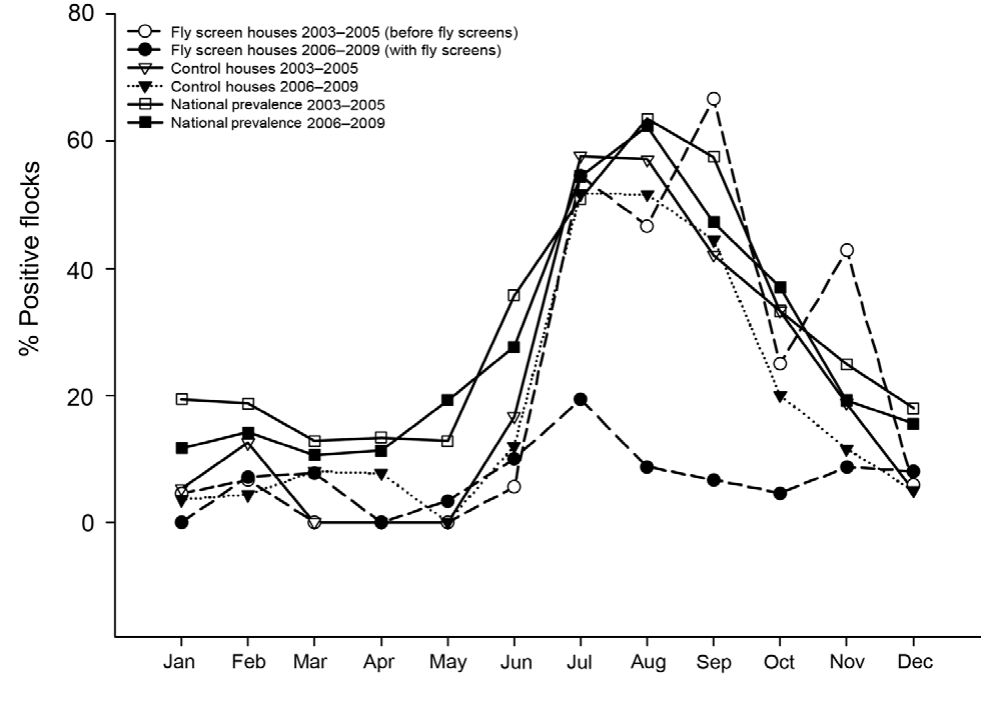
Year-round percentage, by month, of broiler chicken flocks that were *Campylobacter* spp. positive during 2003–2005 (before fly screens) and 2006–2009 (with fly screens).

Seasonal trends in percentage of *Campylobacter* spp.–positive flocks at the fly screen houses (2003–2005, before fly screens) and the control houses (2003–2005 and 2006–2009) were similar to national prevalence trends (2003–2005 and 2006–2009) (Figure 2). Thus, the number of *Campylobacter* spp.–positive flocks increased during June and July and peaked in August and September. However, the number of *Campylobacter* spp.–positive flocks in fly screen houses during 2006–2009 was lower than that in control houses and than that reported nationally during June–October (Figure 2). During winter, however, flock prevalence of *Campylobacter* spp. was not reduced for the fly screen houses. In fact, flock prevalence in the fly screen houses did not differ significantly between summer (June–October) and winter (November–May) during 2006–2009 (p = 0.129).

### PAF

Using the results from the fly screen houses (before and after fly screens had been installed), we calculated the PAF for the national prevalence. We estimated that at the national level, 77% of *Campylobacter* spp. positivity would have been prevented during the summer if fly screens had been part of the biosecurity practice on all broiler chicken farms in Denmark. On a yearly basis, PAF was estimated to be 72%.

## Discussion

We found that by using fly screens to prevent flies from entering broiler chicken houses, it was possible to reduce the prevalence of *Campylobacter* spp.–positive flocks from 41.4% to 10.3%. This long-term reduction of prevalence is in accordance with the previous results obtained in the short-term study by Hald et al. (*30*). Prevalence at the control houses and nationally was slightly lower in 2006–2009 than in 2003–2005, a finding that agrees with the general trend in Denmark during this period (*3*). Furthermore, the summer peak in *Campylobacter* spp. flock prevalence observed nationally and in the control houses was absent in the fly screen houses. Summer prevalence at the fly screen houses was equal to the low prevalence levels observed in Denmark during winter. Because only 1 intervention was tested, and because study and control houses were matched thoroughly, the results convincingly attribute the reduction of *Campylobacter* spp. flock prevalence to the use of fly screens. In addition, our results are based on a 4-year dataset, which highlights the robustness of the findings.

We are unaware of any studies that have correlated the abundance of flies with the prevalence of *Campylobacter* spp.–positive broiler chicken flocks. Data from field studies suggest, though, that flies play a linking role in the epidemiology of *Campylobacter* spp. infections by transmitting *Campylobacter* spp. to broiler chickens (*9*,*16*,*17*). In agreement, 1 study found that flies outside broiler chicken houses can carry *Campylobacter* spp. and pass through ventilation systems into the broiler chicken houses (*15*). The year-round and long-term data, obtained by blocking access of flies to broiler houses, indicate that flies are responsible for a major part of the *Campylobacter* spp. positivity among broiler chicken flocks during the peak season, June–October (Figure 2). The results also show that fly screens affected *Campylobacter* spp. prevalence only during summer and not winter. This finding agrees with the role of flies as vectors for the transmission of *Campylobacter* spp. because June–September is when the abundance and growth of flies peak, thus increasing the likelihood of transmission (*11*,*14*). Furthermore, the number of flies per animal on pig and cattle farms peaks in July and August (*14*), concurrent with peak *Campylobacter* spp. prevalence for broiler chicken flocks (*3*). The key to understanding these correlations is probably the ambient temperature and humidity. The study by Guerin et al. in Iceland found that temperature played a major role in the colonization of broiler chicken flocks with *Campylobacter* spp. and assumed that *M. domestica* houseflies played a role in the epidemiology and seasonality of *Campylobacter* spp. colonization (*36*).

According to our findings, if prevalence of *Campylobacter* spp. among broiler chicken flocks can be reduced, as we have demonstrated, on a national level, then this would reduce the number of campylobacteriosis cases in humans caused by consumption of broiler chicken meat. Models have predicted that the expected change in prevalence of *Campylobacter* spp. among humans is proportional to a decline in *Campylobacter* spp. prevalence among chicken flocks (*6*,*37*).

According to the scientific opinion published by the European Food Safety Authority Panel on Biological Hazards in 2011 (*2*), placing fly screens in broiler chicken farms that already had a medium level of biosecurity during the rearing period was the intervention strategy calculated to give the highest risk reduction (50% to 90%) in public health. In agreement, we found that an estimated campylobacteriosis spp. positivity of 77% among flocks during summer on the national level would have been prevented through 2006–2009 if fly screens had been part of the biosecurity practice on all broiler chicken farms in Denmark. Combining the fly screen intervention during the rearing period at the farm level with interventions during the slaughter processing should place a substantial improvement in food safety of broiler chicken meat within reach.

Use of fly screens, or other means of fly control, could be an easy and effective way to reduce the number of cases of campylobacteriosis among humans worldwide. However, the degree of success depends on several factors. In general, broiler chicken houses should be under strict biosecurity, otherwise the chickens could become *Campylobacter* spp. positive by other transmission routes. Ventilation systems would also need to be automated to compensate for the slight pressure drop of the airflow through the screen. Any costs of installation and maintenance could limit the adoption of the method. The cost of fly screens has been calculated to be €0.01– €0.02 per kilogram of chicken meat, which would reduce farmers’ profits (*38*). On the contrary, fly screens could have other beneficial effects; for instance, fly screens could reduce the prevalence of costly poultry diseases carried by flies. Flies are known to carry other poultry pathogens, such as *Salmonella* spp., *E. coli*, *Pasteurella* spp. and avian influenza virus (*21*,*23*,*39*,*40*). However, such relationships need to be further established and validated by future experiments.

In conclusion, fly screens caused a sustained suppressed prevalence of *Campylobacter* spp. among broiler chicken flocks over 4 years during summer; no seasonal variation was found between summer and winter prevalence among chicken houses with fly screens. Therefore, because the association between *Campylobacter* spp. prevalence among flocks and human health risk has been shown to be linear, fly screens or other equally effective fly control measures might have a substantial reduction effect on the incidence of campylobacteriosis among humans.

Technical Appendix Description of broiler chicken houses.
