# Supplementary material for: Foodborne Disease Prevention and Broiler Chickens with Reduced Campylobacter Infection
Source: Emerg Infect Dis. 2013 Mar;19(3):425–30. doi: 10.3201/eid1903.111593 (PMC3647641; doi:10.3201/eid1903.111593)
Supplement: Technical Appendix — Description of broiler chicken houses. [file 11-1593-Techapp-s1.pdf]

Publisher: CDC; Journal: Emerging Infectious Diseases
Article Type: Research; Volume: 19; Issue: 3; Year: 2013; Article ID: 11-1593
DOI: 10.3201/eid1903.111593; TOC Head: Research

Article DOI: http://dx.doi.org/10.3201/eid1903.111593

Foodborne Disease Prevention and Broiler Chickens with Reduced *Campylobacter* Infection

Immediately after the study by Hald et al. was completed, a construction company was obliged to remove the fly nets in due time before the next flock (*1*). However, after finishing the removal from the 10 houses, it was decided to leave the screens in continuous function year-round on the 10 remaining houses. These 10 houses have been included in the present study. All 10 study farms were one or two (non-windowed) house farms, where flocks had shown a summer peak in *Campylobacter* spp. prevalence and low winter prevalence in the years of 2003-2005. All farmers complied with strict hygiene procedures using dedicated footwear and clothes, and washing hands before entry into and out of the broiler chicken house. All farmers practiced the all-in all-out system, where houses were emptied, cleaned, disinfected and dried between each broiler chicken rotation. The houses were all negative pressure ventilated through 177 - 200 wall inlets type Skov® DA 1000 (Skov A/S, Glyngøre, Denmark) with a capacity of 1080 m^3/h or DA 1200 with a capacity of 1200 m^3/h at 10 Pascal negative pressure. Air outlet took place through 13-15 roof outlets on each house and additionally through gable fans when needed. Houses were surrounded by a 3-m zone with short cut grass or gravel. The sizes of the flocks in the study were comparable to the average flock size in Denmark, which is 33.000 broiler chickens per house at placement.

Publisher: CDC; Journal: Emerging Infectious Diseases
Article Type: Research; Volume: 19; Issue: 3; Year: 2013; Article ID: 11-1593
DOI: 10.3201/eid1903.111593; TOC Head: Research

Reference

1. Hald B, Sommer HM, Skovgård H. Use of fly screens to reduce *Campylobacter* spp. introduction in broiler houses. Emerg Infect Dis. 2007;13:1951–3. PubMed http://dx.doi.org/10.3201/eid1312.070488
